# Wellbeing and Stress Coping among Healthcare and Pharmacy Workers: Experiences during the First COVID-19 Lockdown in Lithuania

**DOI:** 10.3390/healthcare10050787

**Published:** 2022-04-23

**Authors:** Kastytis Šmigelskas, Laura Digrytė-Šertvytienė, Gita Argustaitė-Zailskienė, Aušra Griciūtė, Gabrielė Urbonaitė, Irina Banienė, Aurima Stankūnienė, Nida Žemaitienė

**Affiliations:** 1 Department of Health Psychology, Lithuanian University of Health Sciences, 44307 Kaunas, Lithuania; kastytis.smigelskas@lsmuni.lt (K.Š.); laura.digryte@lsmuni.lt (L.D.-Š.); ausra.griciute@lsmuni.lt (A.G.); gabrieleurbonaite95@gmail.com (G.U.); irina.baniene@lsmuni.lt (I.B.); nida.zemaitiene@lsmuni.lt (N.Ž.); 2 Department of Drug Technology and Social Pharmacy, Lithuanian University of Health Sciences, 44307 Kaunas, Lithuania; aurima.stankuniene@lsmuni.lt

**Keywords:** COVID-19, healthcare and pharmacy workers, wellbeing, stress coping, lockdown

## Abstract

(1) Background. During the COVID-19 pandemic, healthcare professionals faced psychological and social challenges in addition to a sharp increase in workload. The aim of this work was to reveal how healthcare and pharmacy workers assessed their wellbeing and the methods of coping they employed to overcome stress during quarantine. (2) Methods. The mixed-method study was conducted between August and October 2020, integrating quantitative (*n* = 967) and qualitative (*n* = 27) strategies. Doctors, nurses, healthcare administrative staff, pharmacy specialists, and other employees of the healthcare system were interviewed retrospectively about their experiences during and following lockdown (March–June 2020). (3) Results. Overall, 38.7% of the respondents reported a decrease in psychological wellbeing, while 23.4% of the respondents reported a decrease in physical wellbeing during quarantine. The healthcare professionals’ narratives identified a shift from nonspecific fears at the beginning of the pandemic to the more concrete fear of contracting COVID-19, of infecting others, and about their loved ones, as well as undifferentiated fear. Multivariate analysis revealed that a subjective decrease in wellbeing was typical in professionals who had had direct contact with patients infected with COVID-19, as well as those with stronger fears, and those who were more likely to employ compulsive distancing and substance use as ways to cope with stress. (3) Conclusions. The results suggest that lockdown had a negative impact on healthcare workers’ wellbeing during the first pandemic wave in 2020.

## 1. Introduction

The world has been coping with the COVID-19 (SARS-CoV-2) pandemic since the first case was reported in China in December 2019. Lithuania reported its first case about three months later, subsequently entering the first wave of the pandemic that prompted a nationwide lockdown of 3 months.

The pandemic has been causing numerous difficulties for various groups of people, with the healthcare community bearing a disproportionately large burden of increased workload, lower work safety, and emotional uncertainty. Messages about extreme psychological effects of the COVID-19 pandemic on healthcare staff have been emerging since the outbreak began. An early study from China identified that a considerable proportion of healthcare workers reported experiencing symptoms of depression, anxiety, insomnia, and distress; especially affected were women, nurses, and front-line healthcare workers directly engaged in diagnosing, treating, or providing nursing care to patients with suspected or confirmed COVID-19 [1]. COVID-19-related fears have also emerged in the recent literature as a concept related to the experience of working in the medical field during the time of this pandemic, which include being afraid of working in COVID-19 wards, being afraid of treating infected patients, and being afraid of infecting other people [2,3].

Later research confirmed the pandemic’s effect on healthcare professionals. A meta-analysis identified that most studies reported a high prevalence of anxiety (30–70%) and depressive symptoms (20–40%); insomnia, burnout, emotional exhaustion, and somatic symptoms were also similarly reported [4]. Pharmacists were also found to be vulnerable in this pandemic, with nearly half of those polled in the United States experiencing secondary traumatic stress and high burnout rates [5]. Healthcare professionals who deal with COVID-19 seem to be under heightened psychological stress and have elevated rates of psychiatric morbidity, resembling the situation during the SARS and H_1_N_1_ epidemics [6]. There has also been a call to study the overall subjective wellbeing of physicians, seeing as it has been linked to burnout and resilience both in and outside of the context of COVID-19 [7].

COVID-19-related stress among health professionals necessitates finding and employing effective coping measures. Cai et al. identified that medical staff used the following coping measures: following strict protective measures, knowledge of virus prevention and transmission, social isolation, positive self-attitude, and social support [8]. Other reports also emphasized the importance of social support for health professionals’ mental health [9]. According to Cabarkapa et al. [10], in this context, the selection and choice of preferred coping mechanisms by health professionals tends to be influenced by personal characteristics such as cultural affiliation and specific health specialty, highlighting the need for more rigorous studies on stress coping as the process through which the individual “manages the demands of the person–environment relationship that are appraised as stressful and the emotions they generate” [11] (p. 19).

Studies have revealed that medical and nursing staff with higher levels of mental health problems were more interested in skills for self-help and showed a more urgent desire to seek help from psychotherapists and psychiatrists [12]. The literature has suggested different strategies to provide mental health services to healthcare professionals during the COVID-19 pandemic [13]. However, the implementation of psychological intervention services sometimes encounters obstacles, as medical staff are reluctant to participate in the group or individual psychology interventions provided to them [14]. Such obstacles might include insufficient time, stigma, fear of professional consequences, professional culture, and poor access to services [15], although information is lacking on what the predictors are in the current pandemic.

Thus, the aim of our study was to shed light on the general mental health condition of healthcare workers and pharmacists during the first COVID-19 lockdown in Lithuania. More specifically, to understand the process and possible outcomes of this experience, we addressed the following objectives: to measure the physicians’ and pharmacists’ exposure to the COVID-19 virus, to assess their pandemic-related fears, to evaluate their stress coping choices, to determine changes in subjective wellbeing, and to analyze the reasons for seeking mental healthcare, as well as the reasons for failing to seek it. We did not hypothesize prior to the study about the findings and potential associations due to the unique global outbreak of a new virus.

## 2. Material and Methods

### 2.1. Study Design and Procedures

The study was conducted from 18 August to 12 October 2020 within the project on COVID-19 pandemic-related job challenges, psychological wellbeing, and support needs in healthcare workers and pharmacy specialists. The study obtained the approval of the Kaunas Regional Biomedical Research Ethics committee (20 July 2020, No. BE-2-88).

To achieve the project’s aim, a mixed parallel design study was chosen, which combined a quantitative and qualitative data collection approach. The design was chosen to examine the phenomena under investigation from a broader and more diverse viewpoint [16,17]. A nonsequential design of the mixed study was used; thus, quantitative and qualitative data were collected simultaneously.

The study involved Lithuanian healthcare workers: medical doctors, nurses, pharmacy specialists, administrative workers, and other staff. Convenient sampling for both the quantitative and the qualitative arm was used. The study tool was provided online to give the target groups equal opportunities to participate regardless of location in the country. The target groups for the quantitative survey were reached by contacting the administration of healthcare settings and pharmacies, as well as sending out survey invitations via institutional emails. On the basis of the official data about healthcare settings in the country, 222 target institutions were reached. Healthcare workers from 56 institutions voluntarily responded to the research questions. For qualitative interviews, participants received invitations during the quantitative survey and were also identified using the “snowball” method. All persons reached were eligible for participation in the study, provided they were working in a healthcare setting or pharmacy at the time of participation and provided informed consent. No payment was provided to compensate for participation.

The interviews were conducted by eight researchers who were members of the project team. Qualitative data were collected through approximately 30–45 min individual semi-structured interviews on the agreed online platforms (“MS Teams” or “Zoom”). The audio of the interviews was recorded given the online or paper approval of the informed consent by study participants.

### 2.2. Study Sample

Overall, 967 healthcare workers participated in the quantitative survey—89.6% women and 10.4% men, mean age 42.7 years. Approximately half of the participants were physicians or nurses, with the rest being pharmacy specialists, administrative staff, and people of other occupations. More than two-thirds of participants worked in the public sector, mostly physicians (92.5%) and nurses (96%). In contrast, 87.8% of pharmacy specialists worked in the private sector. Table 1 shows the key characteristics of the quantitative survey sample.

Qualitative interviews were collected from 27 healthcare workers s, who responded to the invitation and gave consent to participate in the interview, including 74.1% women and 25.9% men, mean age 39.8 years. Of them, 37.1% were physicians, 22.2% were nurses, 22.2% were pharmacists, and 18.5% were workers in administrative positions. The number of the qualitative study participants was confirmed as sufficient when the data saturation was agreed upon.

### 2.3. Measures

To collect quantitative data, researchers compiled a questionnaire on the basis of their expert opinion, other surveys, or questionnaires. Given the then absence of COVID-19-specific and validated tools, the selected items were based on previous research related to the conditions of interest in healthcare professionals. The questionnaire did not comprise a thorough scale of a particular psychological construct; therefore, reliability measures were not calculated. The questionnaire was basically evaluated at face validity, checked within the group of researchers, and piloted in the target group. Exposure to COVID-19 was assessed using questions on whether the study participant had worked with COVID-19-positive patients and had previously been in self-isolation. COVID-19-related fears, worries, and anxiety were assessed using five items, according to the work of Wu et al. [18]: “I was afraid of catching COVID-19”, “I thought that I might not survive if I were to catch COVID-19”, “I felt unable to control the threat of catching COVID-19”, “I was worried that I might infect my loved ones with COVID-19”, and “I was worried that people will avoid my family because of my job”, with responses “yes”, “partly”, or “no”. Furthermore, stress coping during lockdown was evaluated. Given the limited volume of the survey, the possibilities for a detailed assessment of coping were restricted; therefore, the section included 10 methods of stress coping. The methods were selected according to the Lithuanian four-factor stress coping questionnaire [19] and other studies reflective of the experiences of healthcare workers [20]. Use of mental health services was assessed with one question “Did you use the services of mental health professionals during the lockdown?”, with the options *yes/no*. A question about reasons that prevent access to psychological services was also included. Subjective wellbeing was evaluated using two questions to reflect its change from the beginning of the lockdown to the participation in the survey. The study also included sociodemographic questions about participants’ age, gender, family, and occupational indicators.

To collect qualitative data, a semi-structured interview method was chosen to help researchers maintain structure but also to expose the subjective experiences of each study participant. The qualitative interview questionnaire was developed by the group of investigators. The main questions were open-ended, designed to refrain from leading participants in the direction of a possible answer, e.g., “Describe your psychological wellbeing during the lockdown period” or “What helped you the most during this period?”. Complementary questions were asked only after the main open questions for filling the responses of the study participants and to structure the content a little more, i.e., “What moods prevailed in your environment?”, “What helped you relax?”, and “How do you assess the availability of psychological help in your environment?”. After the first two pilot interviews, questions were revalidated at a panel meeting. As limitations did not emerge during the first pilot interviews, it was decided to maintain the original structure of the questionnaire and include the pilot interviews in the final database. The data were collected remotely through online platforms by eight researchers through video calls to ensure epidemiologic safety in the context of pandemic. During the interview, the audio was recorded with the informed consent of the participants. The entire investigation met strict requirements of anonymity and confidentiality, as recordings were deleted after the interviews had been transcribed and depersonalized.

### 2.4. Data Analysis

The analysis of quantitative data was run using the “IBM SPSS 20” statistical package. The significance level was set at *p* < 0.05. Descriptive analysis of the sample characteristics and main variables was conducted first, using means and standard deviations (SD) for continuous variables and absolute numbers (*n*) with proportions (%) for categorical ones. Exploratory factor analysis with principal component analysis and varimax rotation was used to obtain insight into latent factors of stress coping. For factor analysis, the indicators of KMO and Bartlett’s tests were used to assess the appropriateness for factor analysis. The varimax rotation was selected with the assumption of an orthogonal nature of underlying latent coping strategies. To define the risk (or protective) emotional and behavioral factors for the deterioration of wellbeing, a logistic regression analysis was conducted. First, the univariate regression was performed to identify potential factors, before moving to multivariate regression with additional adjustment for age, gender, and work sector. The strength of associations was presented as odds ratios (OR) with 95% confidence intervals.

For the analysis of qualitative data, thematic analysis was conducted using an inductive approach by Braun and Clarke [21]. This method is flexible as both external and deeper aspects of the problem under study can be examined simultaneously [22]. The thematic analysis was conducted in six phases [21]: familiarizing with the data, generating initial codes, searching for themes, reviewing themes, defining and naming themes, and producing the report. To ensure the validity of qualitative research, the following validity criteria specific to qualitative studies were applied [23]: clear exposition of the methods of data collection and analysis, reflexivity, attention to negative cases, and fair dealing.

## 3. Results

### 3.1. Qualitative Results

Qualitative research data showed that fear was an important theme for the study participants, and this enabled the researchers to take a deeper look into fears and anxieties experienced during lockdown. Identified subthemes were (1) non-specified fear, (2) fear of COVID-19 infection, (3) fear of infecting others, (4) fear, anxiety, or worries about the health of loved ones, and (5) anxiety about patients.

1. Non-specified fear (15). Study participants experienced a non-specified feeling of fear, which in rare cases could manifest as panic attacks: *“Well, panic attacks, well, maybe I did have …” (9-G)*. Many participants experienced non-specified fear from the beginning of lockdown; on the other hand, data showed that the feeling of non-specified fear was possibly evolving in the duration of the lockdown. For some participants, the feeling of fear increased during lockdown: *“… my fear intensified halfway through lockdown.” (12-S)*. One participant mentioned that the feeling of fear was gone when *“… the drugs were selected, when everything, everything was aligned to, to, to the slightest, as I say, movement of the hand. Then totally, the fear disappeared …” (15-S)*.
2. Fear of COVID-19 infection (10). One of the participants’ perceived fears was the fear of getting infected themselves: *“… after each test, you would actually sit and wait, is is it positive, or is it negative …” (2-G)*. The participants’ fear of being infected was kept up and strengthened by constant work in a risky environment *“… it was scary that it may be there in the ward, that you may be infected ….” (13-S)* and the feeling of suspense: *“And uncertainty. Because you don’t know how long it will continue.” (17-F)*.
3. Fear of infecting others (7). Participants experienced fear of infecting other people: *“In a way it was always scary not that I would get infected, but that I would [infect someone], God forbid …” (10-G)*. Study participants were especially afraid to infect their relatives: *“… to bring the disease home, I mean to my own parents, family and so on.” (6-G)*, and this situation was often resolved by limiting contacts with the loved ones in a variety of ways: *“And after the lockdown was announced … I moved to another apartment so not to bring back that infection to the family.” (20-F)*.
4. Fear, anxiety, or worries about the health of loved ones (9). For the study participants, worrying and anxiety about the health of their loved ones were common: *“… that fear for family, it was so intense.” (7-G)*. Fear and anxiety were revealed in various forms of the participants’ behavior, e.g., anxious thoughts *“… I was worried if she would protect herself …” (8-G)*, monitoring the relatives’ behavior *“… my girl is very sensitive to this ….” (7-G),* explanations, lessons for relatives, and emotions of joy if their loved ones were safe *“… maybe the thing that you’re glad, that your loved ones are all right.” (27-S)*.
5. Anxiety about patients (11). Participants indicated that they were worried about their patients, and they also observed the anxiety experienced by their colleagues: *“… experiencing anxiety, not so much for themselves as for the patients.” (25-AG)*. During work, participants were worried about patients’ emotional states: *“… they (patients) were, too, disturbed very much …” (6-G)*. Several participants mentioned that they had to be psychologically strong, because they felt responsible for calming their patients: *“In fact, in that sense, you have to be not only strong yourself, but also reassure them.” (17-F)*.

Participants in the qualitative study also mentioned fear or concern with losing their jobs and incomes (4); however, being not sufficiently saturated, this subtheme was not included in the analyses.

**Figure 1:**
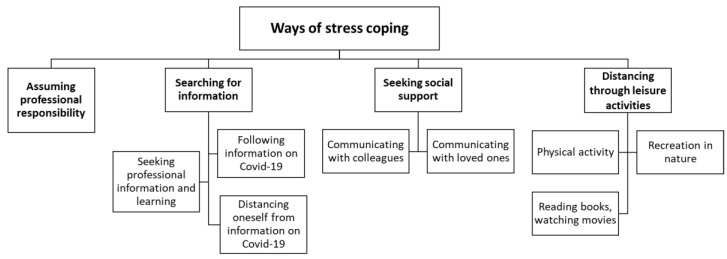
Stress coping among healthcare and pharmacy specialists: thematic analysis.

In the qualitative analysis, four main themes on stress coping emerged: (1) assuming professional responsibility, (2) searching for COVID-19-related information, (3) seeking social support, and (4) distancing through leisure activities (Figure 1).

1. Assuming professional responsibility (15). Faced with the challenges of the COVID-19 pandemic, healthcare workers and pharmacy specialists assumed responsibility and felt a duty to the public in managing the situation. As one participant said, *“… when there are people around who are stressing very much, then you take this other role that you have to manage this whole situation and take responsibility …” (5-G)*. The study participants were able to mobilize in order to deal with the uncertainty, fears, and changed working conditions caused by the COVID-19 pandemic and do their job properly: *“We needed to really pull ourselves together … and there wasn’t time.” (26-AS)*. Despite the encountered difficulties, study participants expressed satisfaction with the work they were doing and were pleased that they were able to reaffirm their choice of profession: *“I was really glad that I was doing this job and I was very happy with myself that I did not get scared” (11-S)*.
2. Searching for information (21). This theme consisted of three subthemes: 2.1. seeking professional information and learning, 2.2. following information on COVID-19, and 2.3. distancing oneself from information on COVID-19.
  2.1. Seeking professional information and learning (10). At the beginning of the pandemic, most study participants read various scientific publications in order to obtain as much professional information and knowledge about COVID-19 as possible. They also listened to reports from colleagues living in countries more affected by the pandemic and looked into the information and recommendations provided by the Ministry of Health in Lithuania: *“… there was this uuh … more of uuh I don’t know sitting at the computer, what should be done in all these cases when patients would arrive and what would need to be done with them.” (4-G)*. In addition, healthcare workers and pharmacy specialists participated in various trainings to obtain the necessary skills to properly fulfill their duties and learn to protect themselves and their patients: *“… there was a lot of training, putting it on, taking it off, how work in the COVID ward would be organized” (26-AS)*.
  2.2. Following information on COVID-19 (10). At the beginning of the pandemic, in addition to the search for professional information, the study participants were actively and quite compulsively searching for any information related to COVID-19. As one of the study participants recounted, *“… in the beginning there was this, that, it’s increasing, it’s increasing, you’re following and following it, and I also liked to switch on the news” (7-G)*. However, some study participants later noted that this behavior had a negative impact on their wellbeing: *“… to the point of back strain [laughs], to unbalanced sleep, because you keep gobbling up the information on COVID …” (8-G)*.
  2.3. Distancing oneself from information on COVID-19 (7). As the pandemic continued, the behavior of the study participants started changing. Some participants noted that their chosen strategy to know as much as possible about COVID-19 was causing tension, anxiety, and fear. As a result, they reduced their intake or completely distanced themselves from information in the media: *“… In the beginning, the flow of information was probably very big, and, and. But somehow, I very quickly sorted out what was important to me, what wasn’t. Umm. And completely stopped following it at one point.” (3-G)*.
3. Seeking social support (19). This theme describes the efforts of study participants to actively seek social support in order to improve and strengthen their wellbeing. It consisted of two subthemes: 3.1. communicating with colleagues, and 3.2. communicating with loved ones.
  3.1. Communicating with colleagues (17). The majority of study participants sought to communicate with colleagues mainly for two reasons. First, they contacted their colleagues for answers to various professional questions relevant at the time: *“… constant consulting, if anything is unclear then you know that you can call any of the colleagues and ask, and they will definitely answer, and help you solve that problem” (7-G)*. The second objective was the pursuit of emotional support, which the study participants achieved in open communication and joking with colleagues: *“Well, I mean, we grumble, we have a laugh, and that’s it [laughs], and you keep going.” (23-AG)*. Such active, comprehensive communication enabled participants to feel the support and understanding of their colleagues: *“During the lockdown we all cared for each other more, were maybe more sensitive, attentive to one another” (2-G)*.
  3.2. Communicating with loved ones (11). During the lockdown period, the emotional support and understanding of family members and other loved ones was important to the participants of the study; therefore, some of them actively communicated and shared their experiences and feelings with their loved ones: *“And we’d somehow call and on Skype, somehow communicated to everyone, talked, somehow consoled and calmed each other …” (2-G)*.
4. Distancing through leisure activities (19). This theme reveals the efforts of study participants to redirect their attention from the situation associated with COVID-19 to pleasant leisure activities. This theme consisted of three subthemes: 4.1. physical activity, 4.2. recreation in nature, and 4.3. reading books, watching movies.
  4.1. Physical activity (12). Some participants used various forms of physical activity to relax, let out emotional tension, and retreat from concerns associated with COVID-19: sporting, running, dancing, cycling, practicing Nordic walking, or just walking a lot. These activities had a positive impact on the wellbeing of participants, as one of the study participants noted: *“But when I started going out for walks, it was better for me” (12-S)*.
  4.2. Recreation in nature (11). The study participants were pleased that, during lockdown, they were allowed to spend their time safely in nature, which was an opportunity they took. Being in nature, in the garden, in the forest, or by the lake, and watching the awakening spring’s nature enabled participants to move away from the tension caused by COVID-19: *“We have of course a homestead out of town … nature is completely wonderful there, there are lakes, I totally relax” (11-S)*.
  4.3. Reading books, watching movies (7). Reading books and watching movies was another form of leisure activity that enabled the study participants to escape from reality for at least a short time and relax: *“… readings, and movie viewings, so that you would forget yourself a little.” (13-S)*.

It is important to note that some of the study participants used to practice some of those leisure activities in the past as well, before the COVID-19 pandemic. However, as they themselves observed, these activities became particularly significant during the pandemic period and had increased value.

In addition to the methods presented and described, the study participants mentioned religion and meditation (4), creative work and household chores (2), taking medicines (2), and sleep (2); however, these subthemes were not sufficiently saturated and, therefore, were not included in the analysis.

Three main themes were identified in the qualitative study data analysis regarding psychological assistance: 5. sufficient information (17), 6. nonuse (12), and 7. persistent need for assistance (12).

5. Sufficient information (17). More than half of participants spoke about the known availability of assistance and the information provided where they could seek for psychological help: *“… information sent by the workplace … and attachments, what can be done to reduce that stress … I believe that psychological assistance has been fully available.” (4-G)*. It was noted that there was more information about available psychological support than pre-lockdown: *“… during lockdown I saw, I saw phone numbers, lines, in some social media, groups … really much more than there was before lockdown.” (6-G)*. Several study participants also reported using psychological assistance: “*I probably called a psychologist more than once.” (12-S)*.
6. Non-use (12). Less than half of participants reported no need to seek psychological help: *“… I would go for it, at the workplace itself, but the thing, I did not need it.” (13-S)*. The study participants also shared their thoughts about the stigmatization of seeking assistance: *“… not in accessibility, but probably in myself. What must happen to me that I would seek it … medical staff often imagine that I am going to handle everything here on my own” (23-AG)*, or the sensitivity to talk to a psychologist working in the same institution: *“I would not dare [to go to a psychologist] in my workplace. Just, there would be some kind of a barrier” (8-G)*. In some cases, communication with colleagues replaced psychological help: *“It was at that time that psychological assistance was as much as we were communicating with each other” (22-F)*.
7. Persistent need for assistance (12). Moreover, fewer than half of the study participants expressed a strong need for psychological help: *“And probably I really wanted the intervention of that professional, I was living in that constant struggle, in the remorse.” (10-G)*, as well as the lack and need of a proactive offering: *“… help would actually have been useful, but to offer—no one around had such an idea. I probably would not have refused because it was a really difficult period.” (16-S)*. The situation could also have been complicated by taking responsibility for the psychological wellbeing of patients: *“We had to be their psychologists because we had to communicate with them [patients] and there was no one to communicate with us.” (15-S)*.

In general, this study revealed that workers in the fields of healthcare and pharmacy used mental health services very rarely, even though the awareness about access to mental healthcare was quite sufficient. Among the main causes for reluctance to use these services were business and a certain level of subjectively perceived stigmatization.

### 3.2. Quantitative Results

Quantitative analysis showed that majority of the participants (74.6%) reported not having worked with COVID-19 patients during the first wave lockdown, while 8.7% worked directly with COVID-19 patients many times. Overall, 13.5% of participants reported having been in self-isolation due to suspected COVID-19 during lockdown; this proportion ranged from 9.3% among pharmacy specialists to 17.9% among physicians.

The quantitative data also revealed high levels of concern about infection; 65.9% of respondents reported fear of infecting their relatives, 41.9% reported fear of getting infected themselves, and 8.5% reported fear of death due to COVID-19. In addition to that, about one-quarter of study participants felt that they had no control over managing the threat of infection (27.1%) and were afraid that other people would avoid their family members due to the participant’s work in healthcare (27.9%). Summarized quantitative data about fear of getting infected are presented in Table 2.

The study participants were asked how they managed to cope with stress during the lockdown period. Quantitative analysis showed that the most common coping strategies employed to combat COVID-19 related stress during lockdown (Table 3) were talking to relatives, friends, or colleagues (75.1%), browsing online (68.4%), and being in nature (66.2%), while the ones used most rarely were drinking alcohol (6.9%) and taking medicine to decrease tension or stress (7%).

**Figure 2:**
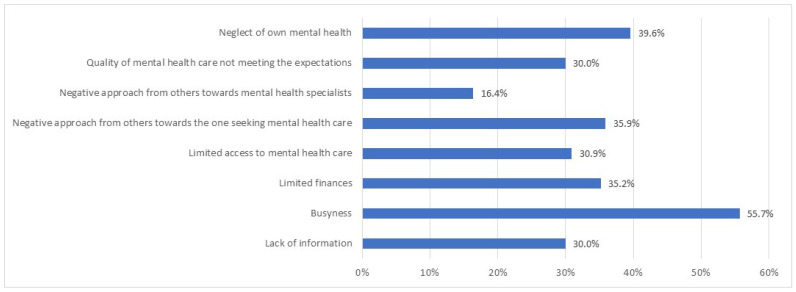
Obstacles to the use of mental health services among health care and pharmacy specialists.

In total, only 5.6% of the participants reported having used mental health services during the pandemic. Among respondents with non-defined occupations, 12.8% used mental health services, while this number ranged from 2.8% to 5.2% among physicians, nurses, pharmacy specialists, and administrative staff. The quantitative survey revealed that the main perceived obstacles for not using mental health services was business (56%) and neglect of mental health (40%), although other causes such as society’s negative view on mental healthcare or limited access to it were also frequently mentioned (Figure 2).

To see the latent strategies of coping, an exploratory factor analysis was conducted (KMO = 0.61, Bartlett’s *p* < 0.001). The varimax rotation revealed three potential factors that were labeled as (1) health-friendly coping (sporting and physical activity; talking to relatives, friends, or colleagues; being in nature), (2) compulsive distancing (browsing online; eating more than usual; sleeping more than usual), and (3) substance use (taking medicine to decrease tension and stress; smoking; drinking alcohol). The qualitative findings partly support and supplement these findings.

In the total sample, 12.9% of quantitative study participants reported their physical wellbeing improving from the beginning of lockdown, while 23.4% reported it deteriorating, and others did not feel any changes. As for psychological wellbeing, 19.7% reported improvement, while 38.7% reported deterioration. To identify the risk and protective factors for the deterioration of wellbeing, a logistic regression analysis was conducted.

In univariate analyses, age, work field, direct work with COVID-19 patients, fear of getting infected, and all three coping strategies (health-friendly coping, substance use, and compulsive distancing) emerged as the most significant predictors of changes in physical and emotional wellbeing. Self-isolation due to suspected COVID-19 and use of mental health services during the lockdown were not significantly associated with the outcomes.

When adjusting for gender, age, and work sector, several findings emerged. Having worked with COVID-19 patients predicted negative changes in physical and emotional wellbeing. Having worked with COVID-19 patients many times was more strongly associated with a negative wellbeing change than working with such patients less frequently. Fear of both getting infected and infecting others was more consistently associated with negative outcomes compared to either only fear of getting infected, or only fear of infecting others.

The final multivariate analysis revealed (Table 4) that compulsive distancing and substance use were consistently associated with subjective wellbeing, increasing the odds for the deterioration of physical (OR 1.26 and 1.49) and psychological wellbeing (OR 1.27 and 1.34). On the contrary, health-friendly coping was found to be a protective factor, showing higher odds for better physical wellbeing (OR = 0.79), while the potential effect on psychological wellbeing was nonsignificant (OR = 0.89).

In summary, negative changes in physical wellbeing during lockdown were predicted by working directly with COVID-19 patients several times, fear of infecting oneself and one’s relatives, compulsively distancing oneself, using substances as a way of coping with stress, and employing less health-friendly coping strategies. Negative changes in psychological wellbeing during the lockdown were predicted by working in the private sector, working directly with COVID-19 patients, fear of infecting oneself, as well as one’s relatives, compulsive distancing, and substance use.

## 4. Discussion

The COVID-19 pandemic in early 2020 influenced a wide range of facets of life, from economic to political limitations, everyday to professional life changes, and personal life to social interactions. The field of healthcare was among the first to react and to deal with the direct consequences of this pandemic. Such a situation revealed that the people providing healthcare to others may suffer greatly themselves, resulting in their reduced capacity or even retreat from profession, which had the opposite impact to what is required; instead of a higher availability of the healthcare workforce under severe circumstances, it may be reduced. As a result, it is important to discuss the challenges that health workers face not only from a physical but also from a mental standpoint. The knowledge about potential risk or protective factors for better wellbeing could further enable the individuals, societies, and systems for change, be it personal or societal.

Our study was launched just after the first wave of COVID-19 in Lithuania during late summer and early autumn 2020. Even though, by this time, Lithuania was among the countries with the lowest infection rates across Europe, our study revealed that almost one in four healthcare workers had had direct contact with COVID-19-positive persons before this study. Moreover, two-thirds of people in our sample reported having a fear of infecting themselves or their relatives, with more fear about relatives than themselves. In addition to that, qualitative data revealed high levels of non-specified fears, especially at the onset of lockdown. We also found that physicians, nurses, pharmacists, and other people providing or acting in field of healthcare had distinct preferences for stress coping; initially, their coping mechanisms were mostly problem-focused, but they later transitioned to emotion-focused strategies such as social support, being in nature, or browsing online. Nonetheless, 23% still reported deterioration of physical wellbeing, while 39% reported deterioration of mental wellbeing, and the strongest predictors of poorer wellbeing were direct contact with infection, fear of infection, and less healthy coping strategies. However, seeking mental health services in our sample was still very rare (below 6%). What could the reasons be for such findings? Are they unique?

Fear is one of the psychological issues that healthcare staff face [24]. Fear mobilizes human energy and enables adaptation in difficult situations [25]; thus, health professionals need more assistance, recommendations, and recovery programs in addition to a greater understanding. Research has shown that fear is a factor that tends to exacerbate a person’s mental health problems. A subjectively perceived high risk of infection, which can indicate having a fear related to it, may make staff feel vulnerable or feel a loss of control over their health [26]. Our findings are in accordance with other COVID-19-related studies showing that fears pertaining to the possibility of infection are related to mental health issues [27], as well as those responding to the call for evaluating the link between such fears and mental health [28]. Our qualitative data revealed non-specified fear in the beginning of the pandemic. Fear-eliciting situations are likely to have become more knowable, predictable and, therefore, more acceptable during lockdown, and later, the workers were better able to specify such fears and alert situations, with the main ones being fear of infecting oneself or others and worrying about relatives’ health and patients. There is a scarcity of studies on such attitudes and dynamics. Practical application of the study data implies more opportunities to provide situation- and needs-based help to healthcare workers during difficult and threatening situations, taking into account the possible changes in the fear response, as well as better understanding of the range of possible manifestation of fear reactions, with the main ones being fear of infecting oneself or others and worrying about relatives’ health and patients.

Other quantitative studies conducted during the COVID-19 pandemic also revealed the fears of infecting oneself or family members [29]. In our study, we found that participants made efforts to deal with and manage the fear-related situations. It should be noted that fear as a reaction does not act as an adaptive function if it dominates or if the person denies feeling it [30].

The qualitative analysis of stress coping in our study identified four main strategies—assuming personal responsibility, searching for information, seeking social support, and distancing through leisure time. One recent qualitative study of emergency healthcare workers found that a passion to help society and country was one of the main strategies related to experiencing COVID-19 stress [31]. In our study, more than half of participants also emphasized the COVID-19 pandemic as a challenge to cope with; this manifested in self-determination, mobilization, a sense of duty, and the wish to reaffirm one’s profession in the face of COVID-19. It is likely the kind of behavior expected from healthcare workers by society; moreover, this willingness to help ill people possibly prompted others to choose the profession. Therefore, some participants had a sense of passion and pride when tackling the COVID-19 pandemic. In contrast, other workers that were not in the first lines of reaction to the pandemic sometimes felt inferior, useless, or “not heroes”. This could be partly explained by the findings of a study in which having a sense of openness to challenges and devotion to work helped oncology professionals to deal with stress at work [32].

It is important to note that study participants applied different ways of coping when dealing with stress and fear of COVID-19, depending on the situation. This was mostly revealed through changes in the search for information on COVID-19. At the beginning of the pandemic, the participants sought to obtain as much information as possible and learn from it. In this way, they tried to make sense of the new situation, fill the knowledge gap, and acquire the necessary skills, such as how to properly protect themselves and others. Such behavior can be described as an application of a problem-oriented stress coping strategy, where a person takes active action to eliminate the influence of stressors [33]. Previous studies have shown that a task-oriented coping style is dominant among physicians [34,35]. This may also be due to the particularities of their work and when many decisions must be made, e.g., diagnosing disease and prescribing treatment. However, for some of participants, this coping strategy became unsuccessful later; they noticed that the flood of information was having a negative effect on their wellbeing and, thus, they tried to minimize it or distance from it. This information-avoiding behavior can be described as one of the ways in which an emotion-oriented stress coping strategy is applied. In such cases, coping strategies are aimed at reducing emotional distress rather than taking specific actions directly to change the stressor [11]. We can assume that, during the pandemic, study participants perceived the COVID-19-related situation as an event that could not be significantly altered; therefore, they were likely to use other emotion-oriented ways of coping.

In addition, it should be noted that most healthcare workers and pharmacy specialists in the study tended to employ several simultaneous methods of overcoming stress rather than just one. Most participants sought social support from both colleagues and relatives. They also distanced themselves from the COVID-19 situation through various forms of leisure, such as physical activity, recreation in nature, reading books, and watching movies. Similar results were obtained in a study involving oncologists [36]. Their most frequently used stress-coping strategies were also maintaining relationships with family and/or friends (69%), reading books and watching movies (66%), emotionally detaching from their problems (63%), and contact with nature (62%) [36]. This suggests that adaptive coping was a frequent option among healthcare professionals during the pandemic.

Our quantitative data showed that direct work with COVID-19-positive patients, fear of infecting oneself and one’s relatives, and frequent substance use and compulsive distancing as ways of coping were related to subjectively deteriorated physical and mental wellbeing. Health-friendly coping was associated with lower risk of deteriorating physical health.

The results are, to some extent, consistent with previous studies. Higher levels of emotional distress and poorer mental wellbeing were seen in healthcare staff who worked directly with COVID-19 patients during the first wave of the pandemic [37,38]. Healthcare workers’ deterioration of physical health and wellbeing has rarely been analyzed in this context. However, there is some evidence that working with COVID-19-positive patients affected the workers’ mental health, which in turn was associated with their worsened subjective physical health perceptions [12]. As the interdependence between mental and physical health is well-established, the pandemic might be offering some insight into the causal relationship between the two in the context of prolonged stress.

Fear of getting infected and infecting one’s relatives was also found to be associated with the subjective deterioration of mental and physical wellbeing in our study. This finding partly echoes the results of other studies on healthcare workers’ mental health during the COVID-19 pandemic [39] and earlier research in the context of severe acute respiratory syndrome (SARS) [40]. Studies also showed that healthcare workers often exhibit more fear of infecting others, e.g., family members [40], which may indicate that the health of their relatives is a concern more closely related to one’s emotional health.

Coping with the stress of a worldwide health threat may be a daunting task. Our study mostly investigated emotion-focused coping strategies and their relationship with wellbeing. The results partly adhere to what is already broadly known of coping mechanisms; distancing oneself from the problem by way of excessive sleeping, eating, and browsing online, as well as using psychoactive substances, is associated with poorer wellbeing and higher emotional distress [41,42]. A noticeable result in our study is that health-friendly coping was not associated with lower risk of deteriorated mental wellbeing. In contrast, previous studies mostly showed that adaptive, health-positive ways of coping such as being in the nature or seeking social support are associated with better mental health and wellbeing outcomes [43,44]; therefore, our result raises the question of whether the COVID-19 pandemic is a different context in which this relationship does not exist, or whether yet unknown variables prevent this relationship.

Both qualitative and quantitative data revealed the minimal use of mental health services. These results are in line with the trends observed in other countries in the context of COVID-19, where medical staff, especially physicians, did not express a strong desire to go for psychological consultations [8,12,45]. Even though the majority of participants in the qualitative study reported there being enough information about assistance, a considerable number of specialists also expressed a need for and lack of proactive assistance. In addition, good practice is already emerging where psychological support is further integrated into the environment of medical staff to care for their mental health or integrated into crisis management across the country [14,45,46]. The study also highlights the need for psychological help and several factors that strengthen the denial of this need: sensitivity of seeking help and desire to conceal this need for help. It is known that stigma associated with mental health limits the need to seek and receive help [47], and, in emergencies such as a pandemic, this may provoke a deterioration of psychological wellbeing, increase in psychological distress, and development of mental health disorders.

It is relevant to acknowledge several limitations of our study. First, the participants were reporting and reflecting about the lockdown experiences and perceptions retrospectively, which could have led to some inaccuracies. It may be suggested that this was more subject to error than to bias, because some participants could have overestimated, while others could have avoidantly neglected a recent experience of extreme working conditions, thus underestimating the potential burden of lockdown. Furthermore, it is important to admit that the factors of coping that were defined in the quantitative analysis are not fully valid for the use across different settings and do not reveal the whole spectrum of stress-coping strategies. The qualitative findings with categories of stress coping should also be used carefully as they come from a relatively small and self-selected sample.

Nonetheless, this study also had some advantages and strengths. First, having a relatively large sample and using a mixed method design allowed revealing COVID-19 experiences with an open-minded approach. In analyzing the healthcare field, pharmacists were also included, as they are frequently omitted in this field of research, even though they took over a certain burden during the lockdown through health-related services in the pharmacy setting. Additionally, we chose to not employ a particular theory of stress coping; instead, we based our understanding on the perspective of the qualitative study participants in a quite specific field (healthcare) under extreme conditions. This was revealed in the quantitative and especially in the qualitative arm of the study.

## 5. Conclusions

To conclude, it is worth remarking that, in the months of pandemic-related challenges, the healthcare systems faced infrastructural, organizational, and economic difficulties, that were, in a way, like the “near side of the moon”: frequently reviewed, spotlighted, and analyzed across media and in public. However, the “far side” is usually invisible or neglected: the people within healthcare and related systems face huge challenges associated not only with their duties and responsibilities but also with their personal matters—daily life, emotions, and wellbeing. Qualitative data revealed a more specific understanding of fear manifestation and changes experienced by healthcare workers, from the non-specified fear at the beginning of the pandemic to more concrete and predictable fear of specific situations during the lockdown. This study revealed a quite high prevalence of unfavorable conditions and behavior patterns among healthcare and pharmacy workers, which suggests the need for special attention and care about the people who are saving lives during the pandemic. Our results highlight a discrepancy between the portion of the sample reporting a deterioration of mental wellbeing and those who sought mental health services. For this, the awareness of, attitudes to, and availability of mental health services may be one of the key points in tackling such extreme unavoidable conditions. In addition, training the personal management of one’s mental state could also be beneficial to prevent likely resignations and decreased work efficacy among healthcare professionals.

## Figures and Tables

**Table 1:** Main characteristics of quantitative survey sample.

Indicator	Value	*n*	%
Gender	Women	857	88.6
	Men	101	10.4
Age	Years, mean ± SD	42.7 ± 12.7	
Work field	Public	669	69.2
	Private	267	27.6
Healthcare level	Primary	172	17.8
	Secondary	200	20.7
	Tertiary	325	33.6
	None	206	21.3
Specialty	Physicians	252	26.1
	Nurses	253	26.2
	Pharmacy specialists	245	25.3
	Administrative staff	73	7.5
	Others	130	13.4

**Table 2:** Fear of getting infected with COVID-19.

Type of Fear	*N*	%
None	311	32.2
Oneself but not relatives	64	6.6
Relatives but not oneself	281	29.1
Both oneself and relatives	311	32.2

**Table 3:** Coping with COVID-19-related stress during lockdown (frequently or very frequently).

Type of Coping	*N*	%
Sporting and physical activity	326	36.3
Meditation or praying	179	20.0
Talking to relatives, friends, or colleagues	680	75.1
Being in nature	604	66.2
Browsing online	618	68.4
Eating more than usual	225	24.8
Sleeping more than usual	223	24.8
Taking medicine to decrease tension and stress	63	7.0
Smoking	100	11.2
Drinking alcohol	61	6.9

**Table 4:** Predictors of subjective wellbeing decrease: multivariate logistic regression.

Indicator	Value	Physical Wellbeing		Psychological Wellbeing	
		OR (95% CI)	*p*	OR (95% CI)	*p*
Gender	Men	1.00		1.00	
	Women	1.02 (0.61–1.73)	0.928	0.89 (0.57–1.40)	0.627
Age	Year	0.99 (0.98–1.01)	0.311	1.01 (1.00–1.02)	0.192
Work sector	Public	1.00		1.00	
	Private	1.31 (0.91–1.89)	0.148	1.38 (1.00–1.90)	0.049
Work with COVID-19 patients	No	1.00		1.00	
	Several times	1.58 (1.03–2.42)	0.035	1.49 (1.02–2.17)	0.041
	Many times	1.71 (0.99–2.96)	0.055	2.10 (1.26–3.50)	0.004
Fear of infecting	None	1.00		1.00	
	Oneself but not relatives	1.24 (0.55–2.79)	0.602	1.36 (0.72–2.59)	0.343
	Relatives but not oneself	1.38 (0.88–2.16)	0.166	1.50 (1.03–2.19)	0.035
	Oneself and relatives	1.79 (1.16–2.77)	0.009	2.12 (1.46–3.07)	<0.001
Coping strategies					
Health-friendly	Mean score	0.79 (0.64–0.98)	0.029	0.89 (0.75–1.07)	0.205
Compulsive distancing	Mean score	1.49 (1.22–1.83)	<0.001	1.27 (1.06–1.52)	0.010
Substance use	Mean score	1.26 (1.02–1.56)	0.032	1.34 (1.10–1.64)	0.003

## Data Availability

All databases are available from the corresponding author upon reasonable request.
